# Cost-effectiveness evidence for strategies to promote or support breastfeeding: a systematic search and narrative literature review

**DOI:** 10.1186/s12884-020-03460-3

**Published:** 2020-12-03

**Authors:** Elizabeth M. Camacho, Hannah Hussain

**Affiliations:** Manchester Centre for Health Economics, School of Health Sciences, Faculty of Biology, Medicine, and Health, University of Manchester, Oxford Road, Manchester, UK

**Keywords:** Breastfeeding, Cost-effectiveness, Literature review

## Abstract

**Background:**

Global health policy recommends exclusive breastfeeding until infants are 6 months. Little is known about the cost-effectiveness of breastfeeding promotion strategies. This paper presents a systematic search and narrative review of economic evaluations of strategies to support or promote breastfeeding. The aim of the review is to bring together current knowledge to guide researchers and commissioners towards potentially cost-effective strategies to promote or support breastfeeding.

**Methods:**

Searches were conducted of electronic databases, including MEDLINE and Scopus, for economic evaluations relevant to breastfeeding, published up to August 2019. Records were screened against pre-specified inclusion/exclusion criteria and quality was assessed using a published checklist. Costs reported in included studies underwent currency conversion and inflation to a single year and currency so that they could be compared. The review protocol was registered on the PROSPERO register of literature reviews (ID, CRD42019141721).

**Results:**

There were 212 non-duplicate citations. Four were included in the review, which generally indicated that interventions were cost-effective. Two studies reported that breastfeeding promotion for low-birth weight babies in critical care is associated with lower costs and greater health benefits than usual care and so is likely to be cost-effective. Peer-support for breastfeeding was associated with longer duration of exclusivity with costs ranging from £19–£107 per additional month (two studies).

**Conclusions:**

There is limited published evidence on the cost-effectiveness of strategies to promote breastfeeding, although the quality of the current evidence is reasonably high. Future studies should integrate evaluations of the effectiveness of strategies with economic analyses.

## Background

The World Health Organisation (WHO) recommends that all infants are breastfed exclusively for the first 6 months of life [1]. The cost burden of not breastfeeding is estimated to represent 0.49% of world gross domestic product [2]. The global prevalence of exclusive breastfeeding in 2015 was estimated at 43% [3]. It is a priority for global health policy to increase breastfeeding rates.

There have been a number of systematic reviews (including Cochrane reviews) which suggest that a range of interventions may be successful in increasing breastfeeding rates (e.g. [4–6]). The most recent Cochrane review of ‘breastfeeding in healthy mothers and healthy babies’ reported that all forms of extra support showed a decrease in cessation of ‘any breastfeeding’ and a decrease in cessation of exclusive breastfeeding at 6 months, although noted that the evidence was of moderate quality due to very high heterogeneity [5]. Although there is a growing body of research exploring return on investment and cost burden of not breastfeeding [2, 7, 8], less is known about the cost-effectiveness of specific breastfeeding promotion strategies.

Economic evaluations compare the costs and benefits of different strategies with the aim of estimating which is more likely to be cost-effective (i.e. the lowest cost per unit of benefit). Healthcare budgets are finite and policy-makers need this type of evidence to inform their decisions on how best to allocate these limited resources. To support decision-making on how best to allocate funds for the promotion of breastfeeding, knowing which interventions are most likely to be cost-effective is important.

The aim of this review was to identify, bring together, and critically appraise published evidence regarding the cost-effectiveness of strategies for the promotion and support of breastfeeding. This included exploring characteristics of potentially cost-effective interventions and identifying gaps in current knowledge and potentially important directions for future research.

## Methods

A systematic literature search and narrative review of the findings was carried out to identify robust economic evidence relating to any interventions which support and/or promote breastfeeding. The research questions addressed were:

1. Which breastfeeding strategies are likely to be cost-effective for supporting/promoting breastfeeding?
2. What should be the focus for future research, based on current literature and knowledge gaps?

To improve the chances of identifying all relevant economic literature, the search was broad and included any type of breastfeeding interventions (i.e. not restricted by targeted population sub-group). Some studies publish data on costs related to interventions without conducting a full economic evaluation (i.e. comparing the costs and benefits of more than one strategy) or an incremental cost-effectiveness analysis. The decision was made to only include studies which reported incremental cost-effectiveness analyses because incremental analysis enables meaningful comparison between strategies (e.g. by policy-makers) and is considered to be a robust approach to economic evaluation related to health and healthcare [9].

Studies were required to meet all of the following explicit inclusion criteria: (a) studies focusing on promoting or sustaining breastfeeding, (b) any intervention type, (c) interventions aimed at women, babies, partners, or general population (e.g. public health campaigns), (d) standard or usual care/treatment, an alternative intervention, or a placebo intervention as the comparator, and (e) assessment of incremental cost-effectiveness.

Studies were excluded if they were at least one of the following: (a) previous literature reviews (although screening for potential additional references was conducted), (b) conference abstracts, protocols, and feasibility/pilot studies, or (c) studies focussing on reducing the transmission of HIV/AIDS via breastfeeding rather than promoting breastfeeding directly.

Electronic databases of published literature were searched: MEDLINE, Scopus, National Health Service (NHS) Economic Evaluation Database, NHS Health Technology Assessment Database. The searches were conducted in August 2019. Publications before January 2000 were not searched to exclude older references which do not reflect current knowledge/practice and so are less useful for decision making. The searches were not restricted by language. Search terms focussed on words related to breastfeeding and health economics. The search strategy for each database can be found in Supplementary Material (Table S1). To increase the chances of capturing all relevant publications, the bibliographies of previously published literature reviews were also screened [4–7, 10–22].

Both authors independently evaluated the abstracts of identified studies against the inclusion and exclusion criteria. Full texts were then examined separately by both authors to determine which publications should go through to the data extraction stage. The authors compared their results and resolved any disagreements through discussion until a consensus was reached. The details of publications excluded following full text review are reported in Supplementary Material (Table S2).

Based on guidance from the NHS economic evaluation database handbook, a process of structured data extraction and quality assessment was carried out [23]. Extracted data were reported as descriptive summaries; as this is a narrative review, no statistical tests were conducted on the extracted data. A single form which incorporated both data extraction and quality assessment was designed a priori (by author 1). This was applied to each full text included in the review to obtain data required for the review which included: study methodology, results, limitations, evidence gaps, and quality of methods and reporting (see Supplementary Material, Table S3). An additional tool was also used for quality assessment of included publications, an adapted version of the Consensus Health Economic Criteria (CHEC) list [24]. One author (author 1) completed the data extraction process and a second (author 2) reviewed the extracted data.

Costs reported in currencies other than Pounds Stirling (£) were converted based on the average market exchange rate for the cost year used in the publication [25]. The purchasing power parity was not used for currency conversion as one study only reported costs that had already been converted to US dollars (from Ugandan shillings) and it was unclear which exchange rate had been used for this conversion. Costs were inflated to 2018/19 based on the Hospital and Community Health Services (HCHS) index [26]. The rates used to convert currencies and inflate costs are reported in Supplementary Material (Table S4).

The review protocol was registered on the PROSPERO register of literature reviews (ID, CRD42019141721).

## Results

**Fig. 1:**
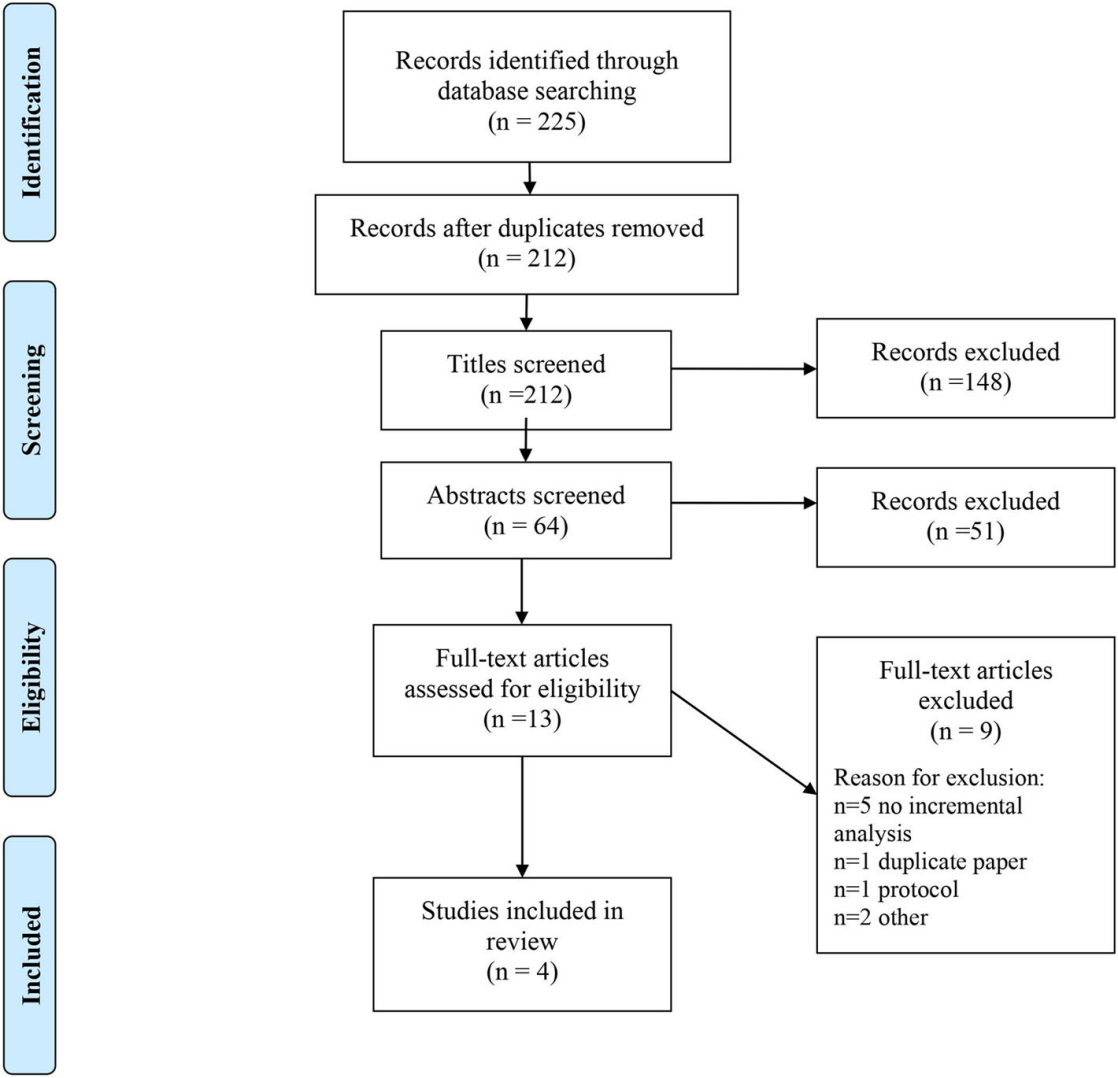
PRISMA flow diagram

There were 225 citations identified from the primary literature searches, 212 remained following deduplication of records (see Fig. 1 for PRISMA flow diagram). The full texts of thirteen publications were reviewed, 4 of which satisfied the inclusion criteria for the review [27–30]. One of the full texts included in the review duplicated the results from a Health Technology Assessment report [20]. The seventeen previous literature reviews related to breastfeeding were hand-searched and resulted in no additional references [4–7, 10–22].

Key characteristics of the 4 included studies are described in Table 1. Two of the studies were based in Europe (UK and Spain) and evaluated a comparable intervention to support breastfeeding in low birth weight babies in a critical care setting through enhanced staff contact with parents [28, 29]. The Spanish study was a replication of the UK model, with adjustment of some of the model parameters which were specific to Spain (e.g. Spanish-specific unit costs). The other two studies evaluated similar interventions (peer/lay support for breastfeeding) and were based in African countries (South Africa [27] and Uganda [30]). The South African study focused on the sub-group of pregnant women who were HIV positive.

**Table 1:** An overview of the studies which were included in the review (*n* = 4)

First author (year of publication)	Population	Country	Intervention (studies reported usual or routine care as the comparator unless otherwise stated)
Desmond (2008) [27]	Pregnant women (sub-group by HIV status)	South Africa	Complex intervention including (full implementation): group education at antenatal clinics, up to 4 antenatal home visits by a lay breastfeeding counsellor, 14 postnatal home visits. Three levels of implementation considered: full, simplified^a^, and basic^b^. Additional comparisons made between levels of implementation of intervention
Rice (2010) [28]	Mothers of babies with low birth weight in neonatal units	United Kingdom	Enhanced staff contact to promote breastfeeding in a neonatal unit
Rubio-Rodríguez (2012) [29]	Mothers of babies with low birth weight in neonatal units	Spain	Intensive promotion of breastfeeding in low birth weight babies
Chola (2015) [30]	Pregnant women	Uganda	Community-based peer counselling conducted alongside breastfeeding promotion in facility-based maternal and child health services, including antenatal and postnatal services. Comparator was facility-based promotion only.

^a^Simplified implementation: Less frequent pre-and post-natal visits, and more clinic based as opposed to home-based visits

^b^Basic implementation: This scenario is entirely clinic-based

*HIV* Human immunodeficiency virus

The study designs are summarised in Table 2. Three out of the four studies reported cost-utility analyses (CUA) [28–30]; the remaining study, which was also the oldest, reported only a cost-effectiveness analysis (CEA) [27]. The two European studies reported the measure of health utility as quality adjusted life years (QALYs) from the perspective of the baby and applied a lifetime horizon [28, 29]. The study from Uganda reported disability adjusted life years (DALYs) based on the health impact on infants over a lifetime horizon and months of breastfeeding over a 6 month horizon [30]. The study from South Africa reported costs and benefits (months of breastfeeding) for a 12 month period [27]. All of the studies were model-based analyses, although an explicit model structure was not reported in the South African study as this was reported as more of a mathematical model [27]. This study was also the only one not to report having conducted probabilistic sensitivity analysis. Only the Spanish study reported a measure of variance around their estimate of net cost e.g. 95% confidence interval [29].

**Table 2:** An overview of the design of the studies which were included in the review (*n* = 4)

First author (year of publication)	Evaluation type	Measure of health benefit	Evaluation details	Data source	Quality/bias considerations
Desmond (2008) [27]	CEA	Month of exclusive breastfeeding	• Trial or model: model (unspecified type) • Perspective: Health service • Time horizon: 12 months • Price year: 2007 • Currency: US $	Patient-level data from an RCT	The comparator used against the basic scenario was base case, whereby no costs were associated. The structure of the model isn’t explicitly described. One-way sensitivity analyses were conducted exploring impact of intervention on breastfeeding and differing staff management scenarios.
Rice (2010) [28]	CUA	QALYs	• Trial or model: model (decision tree) • Perspective: Health service • Time horizon: lifetime • Price year: 2006 • Currency: British £	Published studies/meta-analyses, review of clinical literature	Probabilistic sensitivity analyses were conducted.
Rubio-Rodríguez (2012) [29]	CUA	QALYs	• Trial or model: model (decision tree) • Perspective: Health service • Time horizon: lifetime • Price year: 2011 • Currency: Euros	Published studies/meta-analyses, review of clinical literature	One way (discount rate, cost of intervention) Probabilistic sensitivity analyses were conducted.
Chola (2015) [30]	CEA; CUA	Month of exclusive breastfeeding; DALY	• Trial or model: model (decision tree and Markov) • Perspective: Provider • Time horizon: 6 months; lifetime • Price year: 2007 • Currency: US $	Patient-level data from an RCT, published studies/systematic reviews, patient level data, review of clinical literature	One-way (mortality rate, life expectancy, cost of treating diarrhoea, and discount rate) and probabilistic sensitivity analyses were conducted.

*CEA* Cost-effectiveness analysis, *CUA* Cost-utility analysis, *QALY* Quality adjusted life year, *DALY* Disability adjusted life year, *RCT* Randomised controlled trial

The cost-effectiveness results of the four studies are summarised in Table 3. Both evaluations of interventions targeting low birth weight babies in neonatal units concluded that the interventions were likely to be cost-effective over a lifetime horizon as they were associated with lower costs and QALY gains compared with usual care [28, 29]. There was no single conclusion for the peer support intervention in South Africa but rather the authors concluded that the different derivatives of the intervention (based on level of implementation) could be cost-effective under different assumptions, ranging from £19 to £107/additional month of exclusive breastfeeding. The authors who evaluated the peer-support intervention in Uganda concluded that, at over £9000/DALY averted, it was not likely to be cost-effective in comparison to other maternal/child health interventions already being implemented there [30]. However in terms of the cost/additional month of breastfeeding, this was very close to the midpoint (£63) of the range of costs reported for the peer support intervention in South Africa (£58).

**Table 3:** A summary of the results of the economic evaluations from the studies included in the review (n = 4)

First author (year of publication)	Interventions	Net benefit	Net cost^a^	ICER, key conclusions, and uncertainty
Desmond (2008) [27]	Group education plus breastfeeding counsellor (full, simple, and basic iterations of intervention) versus no support	Incremental increase in months of exclusive breastfeeding: Basic versus no support = 22,306 Simple versus basic = 204,644 Full versus simple = 54,997	Full: £11,513,022 Basic^b^: £5,660,543 Simple^c^: £1,646,915	£19–£107/additional month of exclusive breastfeeding. ***Each of the derivatives of the intervention could be cost-effective under differing sets of circumstances.***
Rice (2010) [28]	Enhanced staff contact in neonatal unit versus usual contact	QALYs by birth weight subgroup: 500-999 g = 0.251 1000-1749 g = 0.056 1750-2500 g = 0.009	500-999 g: -£1030 1000-1749 g: -£515 1750-2500 g: -£116	Intervention was dominant in all weight sub-groups. The intervention would no longer be cost-effective if donor milk were allocated exclusively as a supplement to mothers’ milk. ***Likely to be cost-effective.***
Rubio-Rodríguez (2012) [29]	Enhanced staff contact in neonatal unit versus usual contact	QALYs by birth weight subgroup: 500-999 g = 1.75 1000-1749 g = 0.333 1750-2500 g = 0.156	500-999 g: -£23,859 1000-1749 g: -£6282 1750-2500 g: -£3203	Intervention was dominant in all weight sub-groups. The cost of current breastfeeding promotion (usual care) was not included in the model so costs are conservative. ***Likely to be cost-effective.***
Chola (2015) [30]	Peer support plus clinic-based breastfeeding promotion versus breastfeeding promotion only	2 months of exclusive breastfeeding; 0.01 DALYs averted	£116	£58/month of exclusive breastfeeding; £9617/DALY. ***Not likely to be cost-effective.***

*ICER* Incremental cost-effectiveness ratio, *QALY* Quality adjusted life year, *DALY* Disability adjusted life year

^a^Costs have been inflated to 2017/18 and currencies converted to British £; ^b^Basic implementation: This scenario is entirely clinic-based

^c^Simplified implementation: Less frequent pre-and post-natal visits, and more clinic based as opposed to home-based visits

### Critical appraisal

The full results of the quality appraisal of each included publication are reported in Supplementary Material (Table S5). The papers were all high quality, scoring 16 or higher (out of 18) on the CHEC-list [24]. The two peer-support interventions did not explore all important variables in sensitivity analyses [27, 30] and the two interventions based in neonatal units did not discuss the generalisability or applicability of their findings to other settings [28, 29].

## Discussion

There is a very limited evidence base on the cost-effectiveness of strategies to improve breastfeeding rates. Four studies evaluated two intervention types between them: breastfeeding promotion for low-birth weight babies in critical care and peer support for breastfeeding in low/middle-income countries. Breastfeeding promotion in critical care is associated with greater health benefits and lower costs than usual care, therefore it is likely to be cost-effective. The conclusions from the studies on peer support in lower-income settings are less clear and dependent on specific intervention configuration, but both studies reported comparable costs per additional month of exclusive breastfeeding. Although these studies were of reasonably high quality, three out of the four did not report a measure of variance around their results which makes it difficult to gauge the level of uncertainty in their conclusions. The disparity in the volume of evidence regarding the effectiveness compared to the cost-effectiveness of various strategies to promote breastfeeding is profound and more economic evidence is needed. For example, there are no economic evaluations at the general population level in high-income countries.

Previous systematic reviews in relation to the cost-effectiveness of support or promotion of breastfeeding have similarly concluded that there was limited evidence [4–7, 10–22]. In the time after this review was conducted a systematic review of costing studies related to breastfeeding interventions was published [31]. That review focussed on implementation costs, rather than full economic evaluations which explore the trade-off between costs and benefits (i.e cost-effectiveness), as has been considered in this review. The authors noted a high degree of heterogeneity between the studies they identified which limited comparability. None of the recent reviews have produced narrative summaries and presented costs unified to a single year/currency. This review updates and extends existing cost-effectiveness evidence and is presented in a way which could help to inform evidence-based decision-making by policy-makers/commissioners.

As has been noted in previous Cochrane reviews, trials of interventions to promote breastfeeding should incorporate an evaluation of cost-effectiveness as this is an important gap in current knowledge [5, 12]. The lack of economic evaluations of general population breastfeeding strategies in high income settings is particularly striking. Randomised controlled trials should incorporate both trial-based and model-based economic evaluations to increase relevance to decision-makers. The benefits of breastfeeding for infants may be lifelong, hence economic models which consider costs and benefits (in terms of health utility) over a lifetime horizon are particularly important when comparing the cost-effectiveness of breastfeeding interventions to other public health interventions. However, when comparing the cost-effectiveness of different breastfeeding interventions, short-term outcomes e.g. months of exclusive breastfeeding may be more appropriate. Going forward, the use of standardised measures of health benefit in relation to breastfeeding would aid comparisons between interventions.

Another neglected area in current research is measuring the health benefits and economic impacts of breastfeeding from the perspective of parents. For example, it has been estimated that upwards of 80% of the medical savings and deaths prevented through increased breastfeeding are related to improvement in mothers outcomes [32]. Similarly none of the studies included in this review incorporated the indirect costs for mothers due to time spent breastfeeding, which can be considerable [33]. For example, researchers in the United States reported that breastfeeding for 6 or more months was associated with “more severe and more prolonged” loss of earnings compared with breastfeeding for shorter durations or not breastfeeding [34]. This is also a key area for future research.

A key limitation of this review is that it is not possible to quantitatively synthesise the evidence from the studies identified due to both the small number and heterogeneity of the studies. While this is a common occurrence in reviews of economic evaluations at present [35], with an increase in primary research in the area, this would be a good objective for a future review. One potential source of bias in this review was the restriction of searches to published journal articles; inconclusive or negative cost-effectiveness results are less likely to be published in journals than in the grey literature [36]. For example, a report produced by the Maternal and Child Nutrition Programme in England described an economic model estimating costs and benefits of a peer-support programme to promote breastfeeding was excluded as it was not published in a peer-reviewed journal [37]. Another limitation is that although a comprehensive approach, using two different tools, was used to critically appraise the studies included in the review, one of the tools was developed by one of the authors (author 1) and has been used before, however it has not been formally validated. Furthermore the other tool, the CHEC-list [24], awards a point for a particular item only if it is fully met. This approach aims to minimise subjectivity but may not reflect some finer differences between studies, for example when considering a study which has met 90% of a particular item compared with one which has not attempted it at all.

## Conclusions

Existing evidence suggests that breastfeeding support in a critical care setting is likely to be cost-effective. There are clear indications for researchers in the area regarding the need to incorporate economic evaluations and to establish standardised outcome measures related to breastfeeding which will facilitate future evidence synthesis.

## Supplementary Information

**Additional file 1:** Search strategy, reasons for exclusion of full texts, data extraction and quality assessment, and currency conversion and inflation rates.

## Data Availability

All data generated or analysed during this study are included in this published article and its supplementary information files.

## Abbreviations

CEA: Cost-effectiveness analysis
CHEC: Consensus Health Economic Criteria
CUA: Cost-utility analyses
DALY: Disability adjusted life year
HCHS: Hospital and Community Health Services
NHS: National Health Service
NICE: National Institute of Health and Care Excellence
QALY: Quality adjusted life year
WHO: World Health Organisation
