# Supplementary material for: Cost-effectiveness evidence for strategies to promote or support breastfeeding: a systematic search and narrative literature review
Source: BMC Pregnancy Childbirth. 2020 Dec 3;20:757. doi: 10.1186/s12884-020-03460-3 (PMC7712610; doi:10.1186/s12884-020-03460-3)
Supplement: Supplementary file 1 — Additional file 1: Search strategy, reasons for exclusion of full texts, data extraction and quality assessment, and currency conversion and inflation rates. [file 12884_2020_3460_MOESM1_ESM.docx]

**Table S1 - search strategies (primary literature review)**

| MEDLINE | 1. economic evaluation[Title/Abstract] OR economic analys*[Title/Abstract] OR cost analys*[Title/Abstract] OR cost effective* analys*[Title/Abstract] OR cost-effective* analys*[Title/Abstract] OR cost benefit* analys*[Title/Abstract] OR cost utility* analys*[Title/Abstract] OR cost-benefit* analys*[Title/Abstract] OR cost-utility* analys*[Title/Abstract] 2. breastfeeding OR breast feeding OR breastfed OR breastfeed OR bottle feeding OR bottle fed OR bottle feed OR infant feeding OR human milk OR formula milk OR formula feed OR formula fed) AND (promote OR promotion OR initiation OR initiate OR support OR sustain 3. #1 AND #2   Limit #3 to "2000/01/01"[Date - Publication] : "3000"[Date - Publication] |
| --- | --- |
| Scopus | economic evaluation OR economic analys* OR cost analys* OR cost effective* analys* OR cost-effective* analys* OR cost benefit* analys* OR cost utility* analys* OR cost-benefit* analys* OR cost-utility* analys*  AND  breastfeeding OR breast feeding OR breastfed OR breastfeed OR bottle feeding OR bottle fed OR bottle feed OR infant feeding OR human milk OR formula milk OR formula feed OR formula fed  AND  PUBYEAR > 1999 |
| NHS EED/HTA | *Title search*  breastfeeding OR breast feeding OR breastfed OR breastfeed OR bottle feeding OR bottle fed OR bottle feed OR infant feeding OR human milk OR formula milk OR formula feed OR formula fed  *HTA: published from 1 January 2000 to 31 March 2018  *EED: published from 1 January 2000 to 31 March 2015 |

**Table S2 - reasons for exclusion of full texts screened**

| Title | Year | Lead author | Reason |
| --- | --- | --- | --- |
| Breastfeeding Duration, Costs, and Benefits of a Support Program for Low-Income Breastfeeding Women | 2002 | Pugh [5] | Costs and outcomes reported separately but no incremental analysis |
| A Randomized Trial of Two Public Health Nurse Follow-up Programs after Early Obstetrical Discharge | 2003 | Steel O’Connor [6] | Costs and outcomes reported separately but no incremental analysis |
| The cost-effectiveness of a child nutrition education programme in Peru | 2006 | Waters [7] | Intervention did not aim to promote/support breastfeeding |
| Effectiveness of policy to provide breastfeeding groups (BIG) for pregnant and breastfeeding others in primary care: cluster randomised controlled trial | 2009 | Hoddinott [8] | Costs and outcomes reported separately but no incremental analysis |
| Breastfeeding promotion for infants in neonatal units: a systematic review and economic analysis | 2009 | Renfrew [9] | Duplicates analysis reported in a study included in the review (Rice et al [2]) |
| Costs Related to Promoting Breastfeeding Among Urban Low-Income Women | 2012 | Frick [10] | Costs and outcomes reported separately but no incremental analysis |
| Healthy Beginnings Trial Phase 2 study: Follow-up and cost-effectiveness analysis | 2012 | Wen [11] | Protocol |
| Effect of breastfeeding promotion interventions on cost-effectiveness of rotavirus immunization in Indonesia | 2013 | Suwantika [12] | Although intervention aimed to promote breastfeeding, the economic evaluation focuses on the cost-effectiveness of rota-virus vaccination rather than breastfeeding outcomes |
| South-Africa (Goodstart III) trial: community based maternal and newborn care economic analysis | 2017 | Daviaud [13] | Costs and outcomes reported separately but no incremental analysis |

**Table S3 -** Data extraction and quality assessment form

| **Subject of the study** |  |
| --- | --- |
| Intervention(s) |  |
| Comparator(s) |  |
| **Key elements of the study** |  |
| Type of economic analysis |  |
| Study population |  |
| Details of model (if applicable) |  |
| Setting |  |
| Country |  |
| **Clinical evidence** |  |
| Data sources |  |
| **Measures of health benefit** |  |
| Summary measure of health benefit |  |
| Method of utility valuation |  |
| Time horizon |  |
| Discount rate for health benefit |  |
| **Costs** |  |
| Costs included |  |
| Source of resource use data |  |
| Resource use reported separately from costs |  |
| Sources of unit prices |  |
| Currency and price year |  |
| Adjustment for inflation; other adjustments |  |
| Costs excluded |  |
| Time horizon |  |
| Discount rate for costs |  |
| **Analysis of uncertainty** |  |
| *If model:*  exploration of parameter uncertainty |  |
| *If model:* exploration of structural uncertainty |  |
| *All studies:* exploration of alternative subgroups / settings |  |
| **Estimated benefits** |  |
| Total benefit: intervention arm(s) |  |
| Total benefit: comparator arm(s) |  |
| Net (incremental) benefit |  |
| **Estimated costs** |  |
| Total cost: intervention arm(s) |  |
| Total cost: comparator arm(s) |  |
| Net (incremental) cost (intervention versus comparator) |  |
| **Synthesis of benefits & costs, and conclusions** |  |
| Synthesis of benefits and costs conducted (e.g. ICER) |  |
| ICER value |  |
| Probability cost-effective |  |
| Important differences in results for subgroups or sensitivity analyses |  |
| Summary of authors' conclusions |  |
| **Critical review** |  |
| Is the choice of comparator suitably justified? |  |
| *If model:* was the model structure suitable? |  |
| *If model:* was a model schematic presented? |  |
| *If model:* was the model adequately reported? |  |
| Validity of effectiveness data/estimated health benefit |  |
| Validity of estimated costs |  |
| Do the authors discuss the generalisability of their findings? |  |
| Do the authors compare their findings to previous studies? |  |
| Are the authors' conclusions justified? |  |
| **Implications** |  |
| Do the authors describe policy implications of their findings? Are they appropriate? |  |

**Table S4** - Currency conversion and inflation rates applied

|  | **Price year in study** | **Original currency** | **Exchange rate#** | **HCHS year** | **HCHS index**  **(1987/88 = 100.0)** | **HCHS inflation factor to 2016/17*** | **HS index 2016/17 to 2017/18**** |
| --- | --- | --- | --- | --- | --- | --- | --- |
| Desmond (2008) [1] | 2007 | US dollar | 0.50 | 2006/07 | 249.8 | 1.21 | 1.69 |
| Rice (2010) [2] | 2006 | GBP | n/a | 2005/06 | 240.9 | 1.25 | 1.76 |
| Rubio-Rodríguez (2012) [3] | 2011 | Euros | 0.87 | 2010/11 | 276.7 | 1.09 | 1.53 |
| Chola (2015) [4] | 2007 | US dollar | 0.50 | 2006/07 | 249.8 | 1.21 | 1.69 |
| GBP = Great British Pound; United Kingdom pound sterling (£); US = United States  #per 1GBP; *HCHS index 2016/17 = 302.3 **HS index 2016/17 to 2017/18 factor = 1.4  https://www.ofx.com/en-gb/forex-news/historical-exchange-rates/yearly-average-rates/ | | | | | | | |

**Table S5 -** Criteria list for assessment of methodological quality of economic evaluations: Consensus on Health Economic Criteria [14]

|  | Desmond (2008) [1] | | Rice (2010) [2] | | Rubio-Rodriguez (2012) [3] | | Chola (2015) [4] |
| --- | --- | --- | --- | --- | --- | --- | --- |
| 1. Is the study population clearly described? | ✓ | | ✓ | | ✓ | | ✓ |
| 2. Are competing alternatives clearly described? | ✓ | | ✓ | | ✓ | | ✓ |
| 3. Is the economic study design appropriate to the stated objective? | ✓ | | ✓ | | ✓ | | ✓ |
| 4. Is the chosen time horizon appropriate to include relevant costs and consequences? | ✓ | | ✓ | | ✓ | | ✓ |
| 5. Is the actual perspective chosen appropriate? | ✓ | | ✓ | | ✓ | | ✓ |
| 6. Are all important and relevant costs for each alternative identified? | ✓ | | ✓ | | ✓ | | ✓ |
| 7. Are all costs measured appropriately in physical units? | ✓ | | ✓ | | ✓ | | ✓ |
| 8. Are costs valued appropriately? | ✓ | | ✓ | | ✓ | | ✓ |
| 9. Are all important and relevant outcomes for each alternative identified? | ✓ | | ✓ | | ✓ | | ✓ |
| 10. Are all outcomes measured appropriately? | ✓ | | ✓ | | ✓ | | ✓ |
| 11. Are outcomes valued appropriately? | ✓ | ✓ | | ✓ | | ✓ | |
| 12. Is an incremental analysis of costs and outcomes of alternatives performed? | ✓ | ✓ | | ✓ | | ✓ | |
| 13. Are all future costs and outcomes discounted appropriately? | ✓ | ✓ | | ✓ | | ✓ | |
| 14. Are all important variables, whose values are uncertain, appropriately subjected to sensitivity analysis? | 🗶 | ✓ | | ✓ | | 🗶 | |

|  | Desmond (2008) | Rice (2010) | Rubio-Rodriguez (2012) | Chola (2015) |
| --- | --- | --- | --- | --- |
| 15. Do the conclusions follow from the data reported? | ✓ | ✓ | ✓ | ✓ |
| 16. Does the study discuss the generalizability of the results to other settings and patient/client groups? | ✓ | 🗶 | 🗶 | ✓ |
| 17. Does the article indicate that there is no potential conflict of interest of study researcher(s) and funder(s)? | ✓ | 🗶 | ✓ | ✓ |
| 18. Are ethical and distributional issues discussed appropriately? | ✓ | ✓ | ✓ | ✓ |
| TOTAL SCORE | 17 | 16 | 17 | 17 |
| Each criteria met is awarded one point: 15 or greater = high quality, 8-14 = average quality, less than 8 = poor quality.  Item 13 – studies where discounting is not applicable (i.e. time horizon less than one year) have been assumed to meet criteria. | | | | |

References

1. Desmond C, Bland RM, Boyce G, Coovadia HM, Coutsoudis A, Rollins N, et al. Scaling-Up Exclusive Breastfeeding Support Programmes: The Example of KwaZulu-Natal. Belizan JM, editor. PLoS One. 2008;3:e2454.

2. Rice SJC, Craig D, McCormick F, Renfrew MJ, Williams AF. Economic evaluation of enhanced staff contact for the promotion of breastfeeding for low birth weight infants. Int. J. Technol. Assess. Health Care. 2010;26:133–40.

3. Rubio-Rodríguez D. Análisis económico de la promoción intensiva y especializada de la lactancia materna en las unidades neonatales en España Economic analysis of specialised and intensive promotion of breastfeeding in neonatal units in Spain. An. Pediatr. 2012;77:297–308.

4. Chola L, Fadnes LT, Engebretsen IMS, Nkonki L, Nankabirwa V, Sommerfelt H, et al. Cost-Effectiveness of Peer Counselling for the Promotion of Exclusive Breastfeeding in Uganda. van Wouwe J, editor. PLoS One. 2015;10:e0142718.

5. Pugh LC, Milligan RA, Frick KD, Spatz D, Bronner Y. Breastfeeding duration, costs, and benefits of a support program for low-income breastfeeding women. Birth. 2002;29:95–100.

6. Steel O’Connor KO, Mowat DL, Scott HM, Carr PA, Dorland JL, Young Tai KFW. A randomized trial of two public health nurse follow-up programs after early obstetrical discharge: An examination of breastfeeding rates, maternal confidence and utilization and costs of health services. Can. J. Public Heal. 2003;94:98–103.

7. Waters HR, Penny ME, Creed-Kanashiro HM, Robert RC, Narro R, Willis J, et al. The cost-effectiveness of a child nutrition education programme in Peru. Health Policy Plan. 2006;21:257–64.

8. Hoddinott P, Britten J, Prescott GJ, Tappin D, Ludbrook A, Godden DJ. Effectiveness of policy to provide breastfeeding groups (BIG) for pregnant and breastfeeding mothers in primary care: Cluster randomised controlled trial. BMJ. 2009;338:a3026.

9. Renfrew MJ, Craig D, Dyson L, McCormick F, Rice S, King SE, et al. Breastfeeding promotion for infants in neonatal units: A systematic review and economic analysis. Health Technol. Assess. (Rockv). 2009.

10. Frick KD, Pugh LC, Milligan RA. Costs Related to Promoting Breastfeeding Among Urban Low-Income Women. JOGNN - J. Obstet. Gynecol. Neonatal Nurs. 2012;41:144–50.

11. Wen LM, Baur LA, Rissel C, Flood V, Simpson JM, Hayes A, et al. Healthy Beginnings Trial Phase 2 study: Follow-up and cost-effectiveness analysis. Contemp. Clin. Trials. 2012;33:396–401.

12. Suwantika AA, Postma MJ. Effect of breastfeeding promotion interventions on cost-effectiveness of rotavirus immunization in Indonesia. BMC Public Health. 2013;13:1106.

13. Daviaud E, Nkonki L, Ijumba P, Doherty T, Lawn JE, Owen H, et al. South-Africa (Goodstart III) trial: Community-based maternal and newborn care economic analysis. Health Policy Plan. 2017;32:i53-63.

14. Evers S, Goossens M, de Vet H, van Tulder M, Ament A. Criteria list for assessment of methodological quality of economic evaluations: Consensus on Health Economic Criteria The authors thank the following persons for their participation in the Delphi panel. Int. J. Technol. Assess. Health Care. 2005;21:240–5.
